# An Efficient Computational Approach to Characterize DSC-MRI Signals Arising from Three-Dimensional Heterogeneous Tissue Structures

**DOI:** 10.1371/journal.pone.0084764

**Published:** 2014-01-08

**Authors:** Natenael B. Semmineh, Junzhong Xu, Jerrold L. Boxerman, Gary W. Delaney, Paul W. Cleary, John C. Gore, C. Chad Quarles

**Affiliations:** 1 Institute of Imaging Science, Vanderbilt University, Nashville, Tennessee, United States of America; 2 Department of Physics and Astronomy, Vanderbilt University, Nashville, Tennessee, United States of America; 3 Department of Radiology and Radiological Sciences, Vanderbilt University, Nashville, Tennessee, United States of America; 4 Department of Diagnostic Imaging, Rhode Island Hospital, Providence, Rhode Island, United States of America; 5 Alpert Medical School of Brown University, Rhode Island Hospital, Providence, Rhode Island, United States of America; 6 CSIRO Mathematical and Information Sciences, Clayton South, Victoria, Australia; 7 Department of Biomedical Engineering, Vanderbilt University, Nashville, Tennessee, United States of America; 8 Department of Cancer Biology, Vanderbilt University, Nashville, Tennessee, United States of America; University of Manchester, United Kingdom

## Abstract

The systematic investigation of susceptibility-induced contrast in MRI is important to better interpret the influence of microvascular and microcellular morphology on DSC-MRI derived perfusion data. Recently, a novel computational approach called the Finite Perturber Method (FPM), which enables the study of susceptibility-induced contrast in MRI arising from arbitrary microvascular morphologies in 3D has been developed. However, the FPM has lower efficiency in simulating water diffusion especially for complex tissues. In this work, an improved computational approach that combines the FPM with a matrix-based finite difference method (FDM), which we call the Finite Perturber the Finite Difference Method (FPFDM), has been developed in order to efficiently investigate the influence of vascular and extravascular morphological features on susceptibility-induced transverse relaxation. The current work provides a framework for better interpreting how DSC-MRI data depend on various phenomena, including contrast agent leakage in cancerous tissues and water diffusion rates. In addition, we illustrate using simulated and micro-CT extracted tissue structures the improved FPFDM along with its potential applications and limitations.

## Introduction

The passage of paramagnetic or superparamagnetic contrast agents (CA) through brain tissue induces a measurable drop in T_2_- or T_2_^*^-weighted MR signal [1] that forms the basis for dynamic susceptibility contrast (DSC) MRI. When combined with appropriate kinetic models, DSC-MRI can be used to measure hemodynamic parameters quantitatively, such as blood flow, blood volume and mean transit time [2]. This imaging approach relies upon MR signal relaxation enhancement created by CA-induced susceptibility differences between tissue compartments, such as blood vessels and the surrounding extravascular space. The assessment of tumor perfusion parameters using DSC-MRI has proven to be useful for characterizing tumor grade [3]–[9] and treatment response [10]–[14]. Despite its increased use in brain tumor and stroke patients, accurate calculation of perfusion parameters using DSC-MRI relies on two assumptions: 1) a linear relationship, with a spatially uniform rate constant termed the vascular susceptibility calibration factor (k_p_), exists between CA concentration and the measured transverse relaxation rate change [15]; and 2) the blood-brain barrier (BBB) is intact, so that contrast agent remains intravascular and can be treated as a nondiffusible tracer [2]. However, heterogeneous distributions of blood vessels within tissue and the dependence of susceptibility field gradients on vascular geometry may yield spatially variant k_p_ values across tissue. Furthermore, leakage of contrast agent in tumors with BBB disruption causes additional T_1_ and T_2_^*^ shortening with subsequent distortion of DSC-MRI signal profiles [16]–[20]. Improved characterization of these potential confounding factors could shed new insights into the biophysical basis of DSC-MRI signals and direct future improvements in acquisition and post-processing strategies.

In order to better understand susceptibility-based image contrast, several theoretical [21]–[25] and computational models using fixed perturber geometry (e.g., cylinders or spheres) [25]–[32] have been proposed. To address the limited ability of these computational models to represent the complex vascular morphologies in both normal brain and tumors, Pathak et al introduced the Finite Perturber Method (FPM) for simulating susceptibility-based contrast for arbitrary microvessel geometries [33] and evaluating differences in *k_p_* for normal brain and tumor [34]. The FPM uses estimated magnetic field perturbations to calculate MR signal by simulating proton diffusion and phase accumulation using conventional time consuming Monte Carlo methods.

For realistic complex tissues, the MC method needs to track the diffusion of a large number of spins to capture complex structural features, which in turn can increase the computation time. As an alternative, the Bloch-Torrey partial differential equation describing the transverse magnetization can be directly solved using finite difference methods (FDM). This approach has been previously shown to improve the computational efficiency of such simulations [35], [36] and used to explore water diffusion in MRI and to aid the interpretation of diffusion-weighted imaging measures and their dependence on the morphology of biological structures such as those found in tumors.

In this study, we propose to evaluate the combination of the finite pertuber and finite difference methods, termed the FPFDM, as a tool for modeling susceptibility based contrast mechanisms. Such an approach leverages the strengths of the FPM, for computing magnetic field perturbations for arbitrarily shaped structures, and the FDM, for efficiently computing the resulting MRI signal evolution. The accuracy of the FPFDM is validated by comparison to traditional Monte Carlo methods. The potential of the FPFDM to compute DSC-MRI signals arising from realistic three-dimensional cellular and vascular models as well as micro-CT based renal angiograms is demonstrated. Going forward, the FPFDM provides a useful tool with which to investigate the influence of vascular morphology, contrast agent kinetics and extravasation on DSC-MRI signals.

## Methods

In this section, we first describe a new approach for generating more realistic, three-dimensional tissue structures that can be used for the systematic investigation of DSC-MRI signals arising from heterogeneous tissues. We then describe the computation of the appropriate magnetic field perturbations and the associated MR signal evolution, including the influence of water diffusion, using the FPFDM.

### 1. Tissue Structures

Tissue structures consisting of cells, vessels or both were simulated in a 3D volume sampled with N^3^ voxels. The motivation for exploring whether cellular properties influence DSC-MRI data originates from previous reports demonstrating that contrast agent extravasation and compartmentalization around cells can induce measurable and dynamic changes in gradient echo acquired signals [16]–[18], [37]. While spheres have been used extensively for evaluating susceptibility-based contrast mechanisms they poorly represent *in vivo* cellular distribution and shape. In particular, packed spheres intrinsically provide no means for modeling orientation heterogeneity and are unable to achieve cellular densities that approximate those found *in vivo.* To overcome these limitations we explore here the use of randomly packed ellipsoids [38]. Modeling cells as ellipsoids enables the systematic investigation of several features relevant to DSC-MRI including ellipsoid orientation heterogeneity, volume, aspect ratio and higher packing fractions. For completeness, we compare results from randomly distributed spheres, closely-packed spheres on a face centered cubic (FCC) grid and randomly packed ellipsoids.

Typically, randomly oriented cylinders are used to explore susceptibility contrast mechanisms [25]–[32]. More recently, several groups have employed the use of microvascular angiograms in order to better model in vivo conditions [33], [39], [40]. In order to provide a framework that mimics in vivo conditions but also enables the systematic investigation of the influence of vascular features on DSC-MRI data we explored the use of fractal tree based vascular networks [41], [42]. Starting with an initial cylindrical segment representing an arterial vessel, the vascular tree was created using bifurcation at each junction into smaller daughter segments and a target vascular volume fraction (2%) was used to terminate the fractal tree development. At each junction the diameter of each daughter vessels was calculated using Murray’s law [43] and given some degree of randomness along with the branching angles to create tumor-like heterogeneous structures.

All simulated tissue structures were represented by a binary function *V*(*x,y,z*) indicating whether a given point within the simulation volume lies inside or outside the simulated compartments, i.e.:
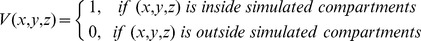
(1)


To further illustrate the versatility of the FPFDM, in addition to the simulated structures, micro-CT was used to create a three-dimensional rendering of a murine kidney vasculature perfused with Microfil (MV–122, Flow Tech). Following perfusion and fixation in 10% neutral buffered formalin, the kidney was scanned in a microCT50 (Scanco Medical AG, Brüttisellen Switzerland). Cross-sectional images of the entire kidney were acquired with an isotropic voxel size of 5.0 µm using an energy of 55 kVp, 200 µA intensity, 700 msec sample time, and 1000 projections per rotation using the manufacturers 1200 mg HA/ccm beam hardening correction algorithm in a 10.24 mm field of view. Using the manufacturer’s software, we assembled individual slices into a z-stack and contrast-filled vessels were segmented from soft tissue by applying a threshold of 260 mg HA/ccm (determined by calibration against a hydroxyapatite phantom) and a three-dimensional Gaussian noise filter with sigma 2.3 and support of 4. The resulting binary three-dimensional reconstruction of the vasculature was then subdivided into MR voxel size sections using in-house Matlab codes (Mathworks, Natick, MA) and used as an input for the FPFDM simulation.

### 2. Computation of Magnetic Field Perturbations

The magnetic field perturbations induced by susceptibility variations within the simulated volume were computed using the FPM [33]. To calculate the magnetic field shift at a given point, the FPM breaks the structure into numerous small cubic perturbers and the contribution to the magnetic field shift due to each perturber is calculated independently. The total magnetic field shift is then evaluated as the sum of the magnetic field shifts from all of the perturbers. As computational input the FPM requires a tabular listing of the vascular/cellular structure *V*(*x,y,z*), the B_0_ field components, and the susceptibility difference (Δχ) between tissue compartments. The magnetic field shift computed by the FPM is:
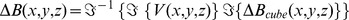
(2)where *V*(*x,y,z*) is a binary tissue structure, 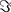 represents the Fourier transform and 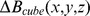 is the magnetic field change arising from a single finite perturber approximated by the magnetic field shift of an embedded sphere within the cube weighted by a volume factor 6/π, and is described by Eq. [3]:
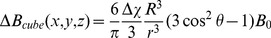
(3)where R is one-half the side length of the cube, Δχ is the susceptibility difference between tissue compartments, and r and θ indicate the distance from the center of the cube and the angle with respect to B0 of the magnetic field calculation point, respectively. The accuracy of this method has been tested using simple geometries with known theoretical field perturbations [33].

### 3. Computation of MR Signal

Estimation of the MR signal relaxation induced by the inhomogeneous field gradients requires simulation of proton diffusion. To track the Brownian motion of thousands of protons over a large number of time steps and calculate their phase accumulation, a Monte Carlo (MC) simulation is frequently used [25]–[31], [33]. The MC method is potentially time consuming for complex tissue structures because in order to accurately calculate the phase distribution it must track a large number of spins that encounter tissue boundaries during their random walks. An alternative approach is to directly solve the Bloch-Torrey partial differential equation using the FDM [35]. The FDM discretizes the tissue sample to a spatial grid and updates the magnetization at each grid point over a series of time steps. To increase the computational efficiency and eliminate edge effects encountered with traditional FD methods we previously developed a matrix-based FDM with a revised periodic boundary condition [36]. For tissue structures sampled with *N* ^3^ voxels the discretized solution of the Bloch-Torrey equation for transverse magnetization (*M*) using the matrix-based FDM is described by:
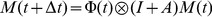
(4)


*A* is an *N* ^3^×*N* ^3^ diffusion transition matrix containing the tissue structural information given in terms of the jump probabilities (probability that a spin starts at one grid point and diffuses to another grid point after a time interval Δ*t*), *I* is an identity matrix with the same size of *A*, and ∶ represents element-by-element vector multiplication. The Φ(*t*) term is a 1×*N* ^3^ vector containing the phase accumulation and relaxation for each voxel at each time step and is given by:
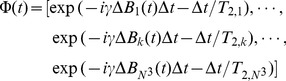
(5)where γ is the proton gyromagnetic ratio (267.5×10^6^ rad s^−1^ T^−1^), Δ*t* is the simulation time step, the subscript *k* indicates a spatial index, 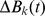 is the field perturbation at point *k*_, and_ *T_2,k_* is the transverse relaxation time at location k within the simulation grid. When a GE sequence is used *T_2,k_* represent the intrinsic tissue T_2_^*^, and in the case of SE it represents the intrinsic *T_2_*. In general, the jump probability from simulation grid *a* to *b* is described by:
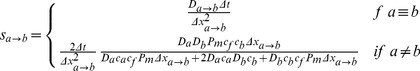
(6)where 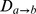 is the diffusion coefficient if a and b are within the same compartment, 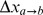 is the distance between simulation grid *a* and *b*, *P_m_* is the permeability of the membrane when a and b are in different compartments, *c_f_* is the free concentration of water. The explicit form of the 1D transition matrix can be found in [36]. The MR signal normalized to the initial magnetization *M^0^* is estimated by summing the magnetizations over all grid points at a particular t and is given by:



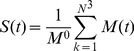
(7)The associated spin echo and gradient echo change in transverse relaxation rates are calculated at a particular echo time *t = TE* using:
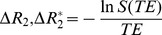
(8)


**Figure 1:**
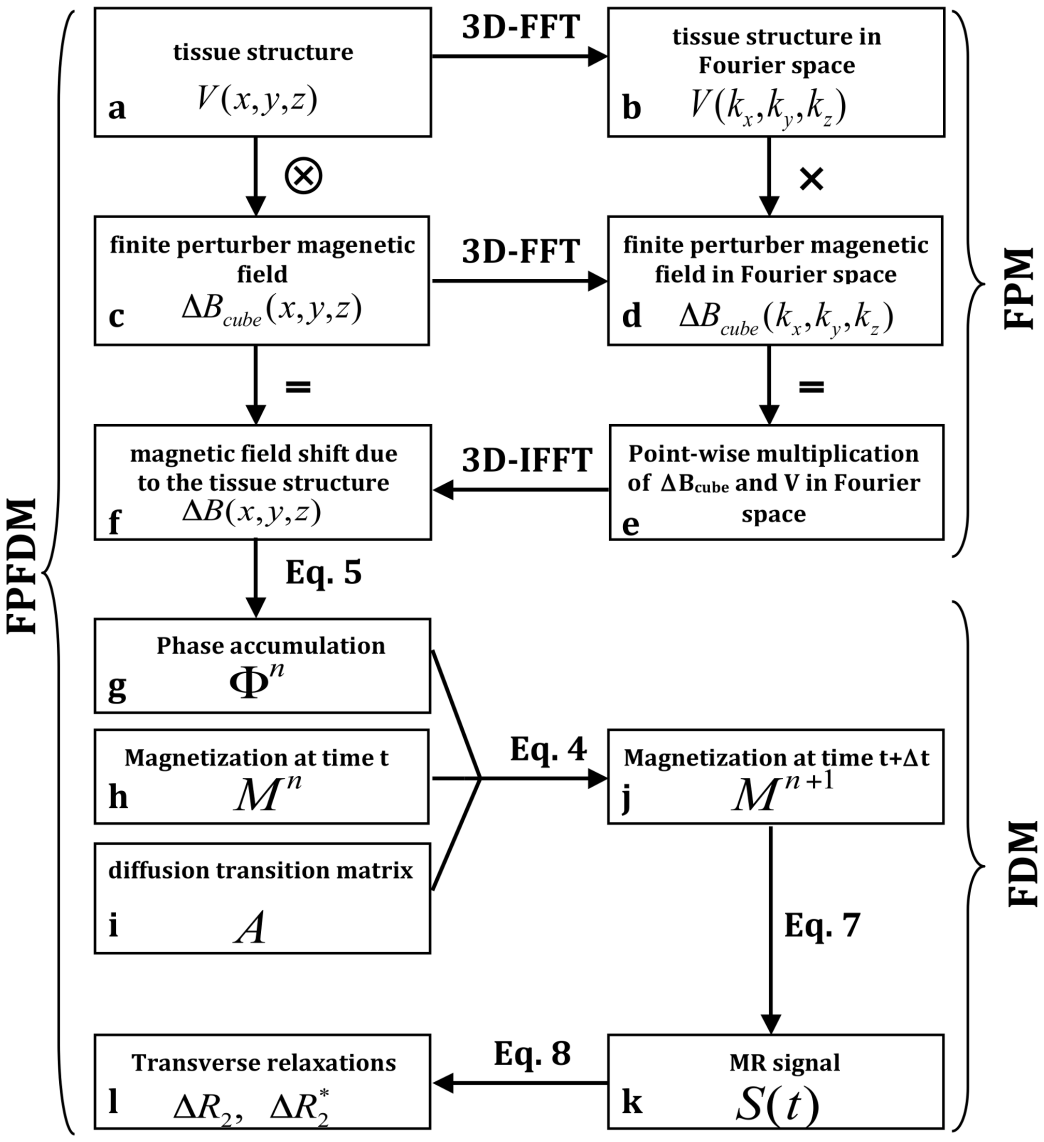
Computational steps involved in the FPFDM. This figure illustrates the steps involved in computing the susceptibility induced transverse relaxation rates for a 3D tissue structure using the FPFDM: (a) The tissue structure is *V*(*x,y,z*). (b) The 3D Fourier transform of (a). (c) The magnetic field from the cubic finite perturber. (d) The 3D Fourier transform of 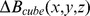. (e) The point-wise multiplication of (b) and (d) in the Fourier domain. (f) The magnetic field shift due to the vascular structure computed as the 3D inverse Fourier transform of (e) or the convolution of (a) and (c). (g), (h) and (i) are the phase accumulation, the magnetization and the diffusion transition matrix, respectively. These are used to compute the magnetization in (j). (k) The computed MR signal. (l) The transverse relaxation rates associated with an arbitrarily shaped tissue structure.

For spin echo imaging, the phase was inverted at *t = TE/2*. This model is designed to handle cases where the three tissue compartments within a voxel can have different intrinsic transverse relaxation times. By updating *T_2,k_* in equation 5, for each grid point at each simulation time step, the total transverse relaxation, which includes the microscopic and mesoscopic relaxation effects, can also be calculated. The decay of signal from large static perturbers is known not to be exponential (e.g. diffusion in a static linear field gradient) but a simple exponential fit is a good approximation for realistic cases, and other functions can be easily fit. All simulations were performed in the Matlab environment (Mathworks, Natick, MA) running on Intel Core 2 Duo at 2.66 GHz and 4 GB of RAM processors. For clarity, the computational steps involved to obtain the final results are illustrated in Figure 1.

### 4. Contrast Agent Kinetics

To demonstrate the potential of the FPFDM to simulate DSC-MRI signals arising from the dynamic passage of contrast agent through the vascular and extravascular spaces, such as would occur in brain tumors with a breakdown of the blood brain barrier, we used tissue structures composed of ellipsoids packed around fractal based vascular network. Concentration time curves were sampled using 150 time points for a total of 9 minutes. The arterial input function (AIF) was generated as previously described [44]. The plasma and extravascular extracellular concentration time curves were computed using the pharmacokinetic two compartmental model described by Brix et al [45]. The relevant input physiological, pulse sequence and physical parameters (e.g. blood flow, blood volume, contrast agent transfer coefficient, *T_1_*, *T_2_*, etc) were selected from values measured in previous MRI, PET and CT brain tumor studies as previously described [16]. To investigate the influence of extravascular features on DSC-MRI, the signal is computed for two cellular structures with a similar cell volume fraction (∼60%) but different ellipsoid radii (5 and 15 µm). The ellipsoids were packed around a fixed vascular tree with a 4% volume fraction.

## Results

### 1. Validation of FPFDM

**Figure 2:**
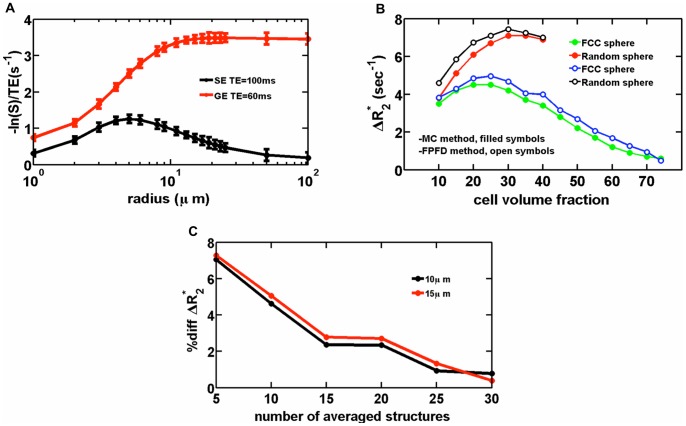
Validation of the FPFDM. (a) FPFDM replicates the characteristic vessel size dependence of ΔR_2_^*^and ΔR_2_ as has been previously shown with MC methods. (b) A comparison of computed ΔR_2_^*^ values as a function of sphere volume fraction and packing arrangement using MC (filled symbols) and FPFDM (open symbols) techniques, with excellent agreement between the two methods. (c) The computed ΔR_2_^*^ percentage difference between MC and FPFDM decreases as the number of FPFDM structures used for averaging increases.

For validation, FPFDM and Monte Carlo based MRI signals were computed and compared for models consisting of randomly orientated cylinders and packed spheres. The dependence of gradient-echo (ΔR_2_^*^) and spin-echo (ΔR_2_) relaxivity on perturber (vessel) size has previously been characterized using Monte Carlo techniques [27]. To replicate these findings we created 10 different structures composed of approximately 40 randomly distributed cylinders for each vessel radius between 1 µm and 100 µm, each with total cylinder volume fraction equal to 2% of the simulation cube. Using the previously reported simulation parameters [27], [33] (susceptibility difference Δχ = 10^−7^_,_ cylinder volume fraction (*V_p_*) = 2%, B_0_ = 1.5T, water diffusion coefficient D = 10^−5^ cm^2^/s, simulation time step Δt = 0.2 ms, GE TE = 60 ms and SE TE = 100 ms), we computed the vessel size dependence of ΔR_2_^*^ and ΔR_2_ averaged over all cylinder arrangements. The computed ΔR_2_^*^ and ΔR_2_ values show negligible changes as the number of averaged structures increases beyond 10. As shown in Fig. 2a, there is excellent agreement between the FPFDM results and those obtained in the Monte Carlo-based comparison studies, which used analytical expressions [27] and FPM [33] for field perturbation calculations.

To compare the computational efficiency of the FPFDM with that of the MC method, we computed ΔR_2_^*^ values using both techniques. For each technique ΔR_2_^*^ values were computed for 18 radii using a TE = 60 ms and Δt = 0.2 ms. The computation time for the FPFDM was approximately 140 seconds per structure. Using 1000 randomly distributed spins, the computation time for the MC method was approximately 220 seconds per structure. Table 1 summarizes the simulation parameters used in the MC and FPFDM along with the respective computational times to generate ΔR_2_^*^ values for 18 cylinder radii.

**Table 1:** Parameters used in MC and FPFDM simulations along with total computing times to calculate ΔR_2_^*^ values for 18 cylinder radii.

Parameters	Meaning	Value
TE	Echo time	60 ms
Δt	Simulation time step	0.2 ms
Δχ	Susceptibility difference	10^−7^
B_0_	Static magnetic field	1.5 T
*V_p_*	Cylinder volume fraction	2%
D	Water diffusion coefficient	10^−5^ cm^2^/s
N_s_	Number of spins used in MC method	1000
Time_MC_	Computing time for MC method	220 s
Time_FPFDM_	Computing time for FPFDM	140 s

To further validate the accuracy of the FPFDM we also computed ΔR_2_^*^ for simulated 3D cellular models consisting of packed spheres. Two packing conditions were considered: randomly distributed spheres and sphere packing on FCC gird. For each model, the sphere size was fixed at 9 µm radius corresponding to an approximate pertuber size where the SE relaxivity peaks and the GE relaxivity reaches plateau [27]. The ΔR_2_^*^ dependence on cell (sphere) volume fraction for the FPFDM was compared to that for the MC method [27] using similar simulation parameters. The MC method was carried out on a different computer system using the approach described previously [27]. Fig. 2b compares the volume fraction dependence of ΔR_2_^*^ for each of the two sphere packing techniques computed by both the MC and FPFDM, using Δχ = 5×10^−8^, B_0_ = 1.5T, D = 10^−5^ cm^2^/s, Δt = 0.2 ms, GE TE = 40 ms, and simulation universe size = (0.5 mm)^3^. The FPFDM results were obtained by averaging the MR signal for 5 different sphere distributions for each packing and cell volume fraction using a simulation grid size of 128^3^. In contrast, the MC method involves tracking 15,000 random walks for each cell volume fraction and the redistribution of the spheres prior to each random walk. The FPFDM results are in excellent agreement to those produced from the MC methodology.

To investigate the convergence of the FPFDM for randomly distributed structures such as those used above, ΔR_2_^*^ values obtained from [27] for vessel sizes of 10 µm and 15 µm were compared to the FPFDM results as a function of the number of structures used for averaging. Fig. 2c shows the percentage difference between the MC and FPFDM derived ΔR_2_^*^ values. For both vessel sizes the computed FPFDM ΔR_2_^*^ values converge to the corresponding reported values [27], [33] to within 7% with only five structure averages. This percentage difference decreases to 0.8% as the number of averaged structures increases to 30.

### 2. Modeling the Effects of Contrast Agent Extravastion on DSC-MRI

**Figure 3:**
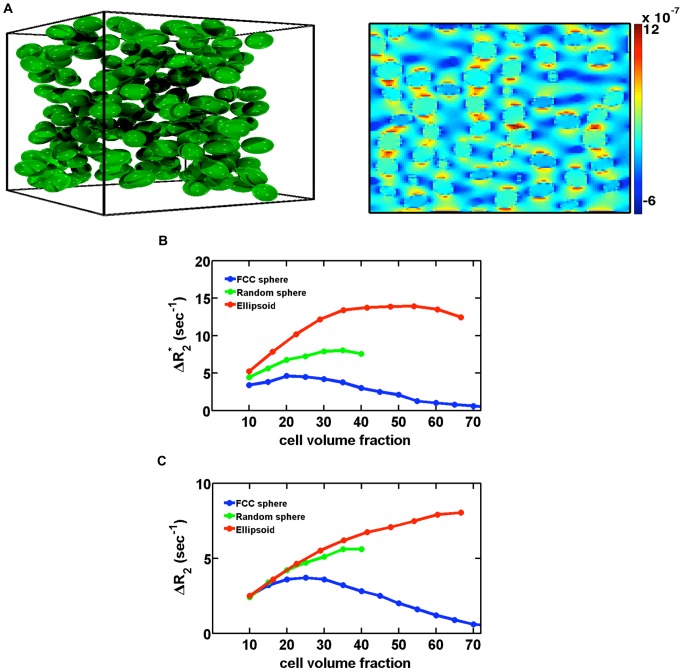
Dependence of ΔR_2_^*^ and ΔR_2_ on cellular shape and packing arrangement. (a) Example of a cellular model using ellipsoid packing (left) and a 2D slice through the associated magnetic field perturbation for B_0_ = 1.5T and Δχ = 5×10^−8^ (right). (b,c) The computed ΔR_2_^*^ and ΔR_2_ dependence on cell volume fraction and packing arrangement. For all packing arrangements, the relaxivity increases and then decreases with cell volume fraction. Ellipsoid packing yields greater relaxivity than spheres. ΔR_2_ exhibits qualitatively similar behavior to ΔR_2_^*^ yet with a reduced magnitude.

To demonstrate the potential of the FPFDM for investigating the complex susceptibility effects that occur when contrast agent extravasates and compartmentalizes around cells, we computed the volume fraction dependence of ΔR_2_^*^and ΔR_2_ for 3D cellular models consisting of packed spheres or ellipsoids (9 µm radius). To model contrast agent leakage effects relevant contrast agent levels corresponding to a Δχ value that is half the peak value of single dose Gd-DTPA injection was assumed. Fig. 3a illustrates the packed ellipsoid model with a representative 2D slice through the computed magnetic field perturbations. Fig. 3b and Fig. 3c demonstrates the influence of packing arrangements on the computed ΔR_2_^*^ and ΔR_2_ values for Δχ = 5×10^−8^, B_0_ = 1.5T, D = 10^−5^ cm^2^/s, simulation time step Δt = 0.2 ms, GE TE = 40 ms and SE TE = 80 ms.

The highly ordered FCC packing of spheres resulted in the smallest relaxivity, reflecting the more homogeneous magnetic field perturbations and proton phase distributions. Randomly distributed spheres yielded slightly greater relaxivities with a non-linear relationship with packing fraction. Finally, the packed ellipsoids, which better approximate cell shape in vivo, enable higher random non-overlapping packing fractions (>65%), are less ordered and also yielded a non-linear relationship between relaxivity and cell volume fraction. For all cell volume fractions, the ΔR_2_^*^ and ΔR_2_ values associated with the ellipsoid-based structures were greater in magnitude than those found with spheres.

### 3. Modeling the Effects of Vascular Tree Heterogeneity on DSC-MRI

**Figure 4:**
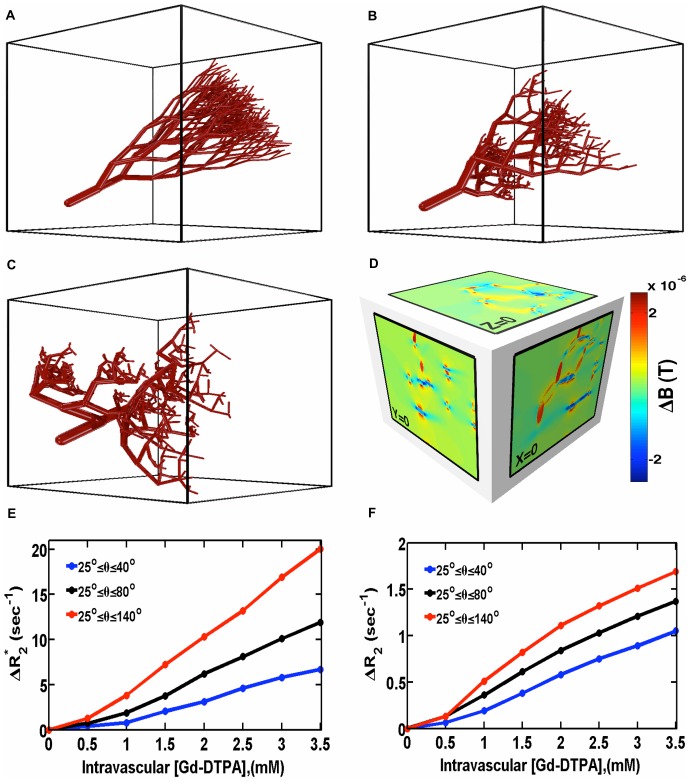
The influence of vascular morphology on ΔR_2_^*^ and ΔR_2_. (a–c) Sample microvascular networks simulated using a fractal tree model with increasing branching angle heterogeneity. (d) Three orthogonal slices through the magnetic field perturbation at the body center for the vascular network in (c). (e–f) Effect of branching angle heterogeneity on the concentration dependence of ΔR_2_^*^ and ΔR_2_ computed with FPFDM (B_0_ = 4.7T, Δχ = 1×10^−7^, 2% target vascular volume fraction). Both ΔR_2_ and ΔR_2_^*^ increase with branching angle heterogeneity.

To illustrate the potential of the FPFDM for modeling the complex geometries of the microvasculature, we used fractal-based branching networks as input to the FPFDM. Fig. 4 illustrates the effect of branching angle heterogeneity (Δθ) on the concentration dependence of ΔR_2_ and ΔR_2_^*^ for typical DSC-MRI contrast agent concentrations. For these simulations we generated three different vascular networks within a 1 mm^3^ volume containing 128^3^ voxels. Fig. 4a–4c shows sample vascular trees with homogenous rotation (φ) angle and bifurcation index (α), which measures the relative diameter of daughter branches at each branching node, with increasing branching heterogeneity (θ). The model for normal vasculature is shown in Fig. 4a, with branching angles ranging from 25°–40°. To represent the tortuous and chaotically organized morphology of tumor vessels, the range of branching angle heterogeneity were increased to 25°–80° (Fig. 4b) and 25°–140° (Fig. 4c). Fig. 4d shows three orthogonal 2D slices through the body center of the magnetic field perturbations computed using the FPM for the vascular structure in Fig. 4c. Fig. 4e and 4f plot the concentration dependence of ΔR_2_^*^ and ΔR_2_ for the three θs considered. For these simulations, Δχ = χ_m_. CA, where χ_m_ is the CA molar susceptibility (0.027×10^−6^ mM^−1^) [46], B_0_ = 4.7T, D = 10^−5^ cm^2^/s, Δt = 0.2 ms, GE TE = 40 ms and SE TE = 80 ms. All simulated vascular structures incorporate vessel radii ranging from 12 µm to 80 µm with a 2% target vascular volume fraction (*v*_p_). The computed relaxation rates were averaged over five different orientations for each simulated vascular network. Using the slope of the ΔR_2_^*^ dose response curves, k_p_ values ranging from 100–295 (mM–sec) ^−1^ were obtained. For this low vascular volume fraction, the k_p_ values for the more tumor-like vascular trees (higher Δθ) were higher than those in normal trees, up to three fold for this simplified simulation. Similar dependency on branching angle with a reduced susceptibility effect was observed for ΔR_2_ dose response curves.

### 4. Modeling First-pass DSC-MRI Data in Brain Tumors

**Figure 5:**
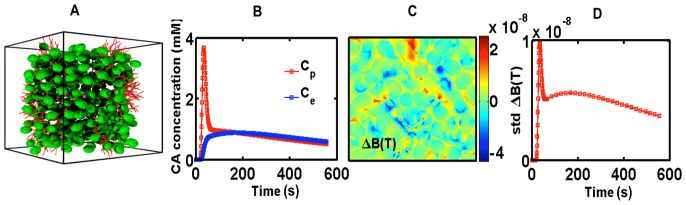
Example simulation with realistic tissue structure and contrast agent extravasation. (a) Sample tissue structure composed of ellipsoids packed around fractal tree based vascular network. (b) Simulated *C*_p_ and *C*_e_ curves derived using 2-compartment model. (c) Example 2D map through the magnetic field perturbation computed at time t = 300 sec. (d). The time evolution of the standard deviation of the field perturbation (std ΔB) computed using B_0_ = 3T, *C*_p_ and *C*_e_ for the given sample structure.

**Figure 6:**
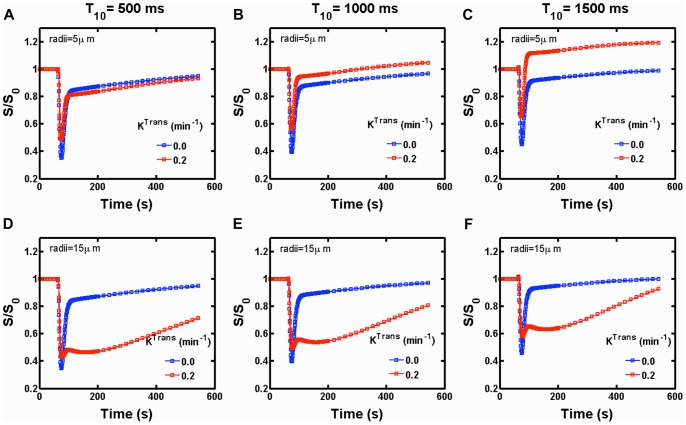
Dependence of DSC-MRI signals on cellular features in the presence of CA leakage. The GE post-contrast to pre-contrast DSC-MRI signal ratio (*S/S_0_*), both in the presence (*K* ^Trans^ = 0.2 min^−1^) and absence (*K* ^Trans^ = 0 min^−1^) of CA leakage at pre-contrast *T*_1_ values of *T*_10_ = 500 ms, *T*_10_ = 1000 ms and *T*_10_ = 1500 ms, for tissue structures constructed using ellipsoids with mean radii of 5 µm (a–c) and 15 µm (d–f), respectively. The (*S/S_0_*) values were computed using input parameters of B_0_ = 3T, D = 1.3×10^−5^ cm^2^/s, Δt = 0.2 ms, TE = 50 ms TR = 1500 ms, α = 90°, T_20_^*^ = 50 ms, r_1_ = 3.9 mM^−1^s^−1^, r_2_ = 5.3 mM^−1^s^−1^ and *P_m_* = 0.

To demonstrate the feasibility of using the FPFDM as a tool to simulate DSC-MRI signals in the presence of contrast agent extravasation, we used two sample tissue structures composed of 60% cells and 4% blood vessels. The two tissue structures were constructed by packing ellipsoids with radii of 5 µm and 15 µm around a fixed fractal-based vascular network. Fig. 5a shows a sample 3D volume rendering of such a tissue structure, which contains ellipsoids of 15 µm average radius and vascular network with vessel size ranging from 5 µm to 45 µm. The simulated vascular (*C_p_*) and extravascular (*C_e_*) contrast agent concentration time curves are shown in (Fig. 5b). Fig. 5c shows a representative 2D slice through the computed magnetic field perturbations at a particular time. The standard deviation of the field perturbation (std ΔB) for all simulated time points is shown in (Fig. 5d). The simulated *C_p_* and *C_e_* curves along with model tissue structure, in Fig. 5, were used as an input to compute the dynamic DSC-MRI signal. Fig. 6 shows the GE post-contrast to pre-contrast DSC-MRI signal ratio time curves (*S/S_0_*), both in the presence (*K* ^Trans^ = 0.2 min^−1^) and absence (*K* ^Trans^ = 0 min^−1^) of contrast agent extravasation. Fig. 6a-c show the time series for the tissue structure composed of ellipsoids with a 5 µm mean radii, at pre-contrast longitudinal relaxation times values of *T*_10_ = 500 ms, *T*_10_ = 1000 ms and *T*_10_ = 1500 ms, respectively. The corresponding time series for the tissue structures modeled with 15 µm cellular radii are shown in (Fig. 6d–6f). The following input parameters were used to compute the DSC-MRI signal: B_0_ = 3T, D = 1.3×10^−5^ cm^2^/s, Δt = 0.2 ms, TE = 50 ms, TR = 1500 ms, flip angle α = 90°, pre-contrast transverse relaxation time T_20_^*^ = 50 ms. The CA T_1_ and T_2_ relaxivity values, r_1_ and r_2_, were set to 3.9 and 5.3 mM^−1^s^−1^, respectively [47]. The compartmental membrane water permeability values were set at *P*_m_ = 0, to model restricted water diffusion. For a fixed cell volume fraction, the simulated time series demonstrate a marked cell size dependency. In general, for both tissue structures, as T_10_ increases from 500 ms to 1500 ms the influence of T_1_ leakage effects becomes more substantial, as indicated by the increased signal recovery. For the small cell size structure, the T_1_ leakage effects eventually result in a signal overshot from baseline (e.g. Fig. 6c). However, the structure constructed with larger cell sizes is dominated by T_2_^*^ leakage effects (as apparent from the low signal recovery well after the CA’s first pass) even at T_10_ = 1500 ms (Fig. 6f). The simulation time to compute the signal for 150 time points, for 3 T_10_ values, 2 contrast agent leakage conditions and 2 tissue structures was approximately 240 mins.

### 5. Application to Image-based Tissue Structures

**Figure 7:**
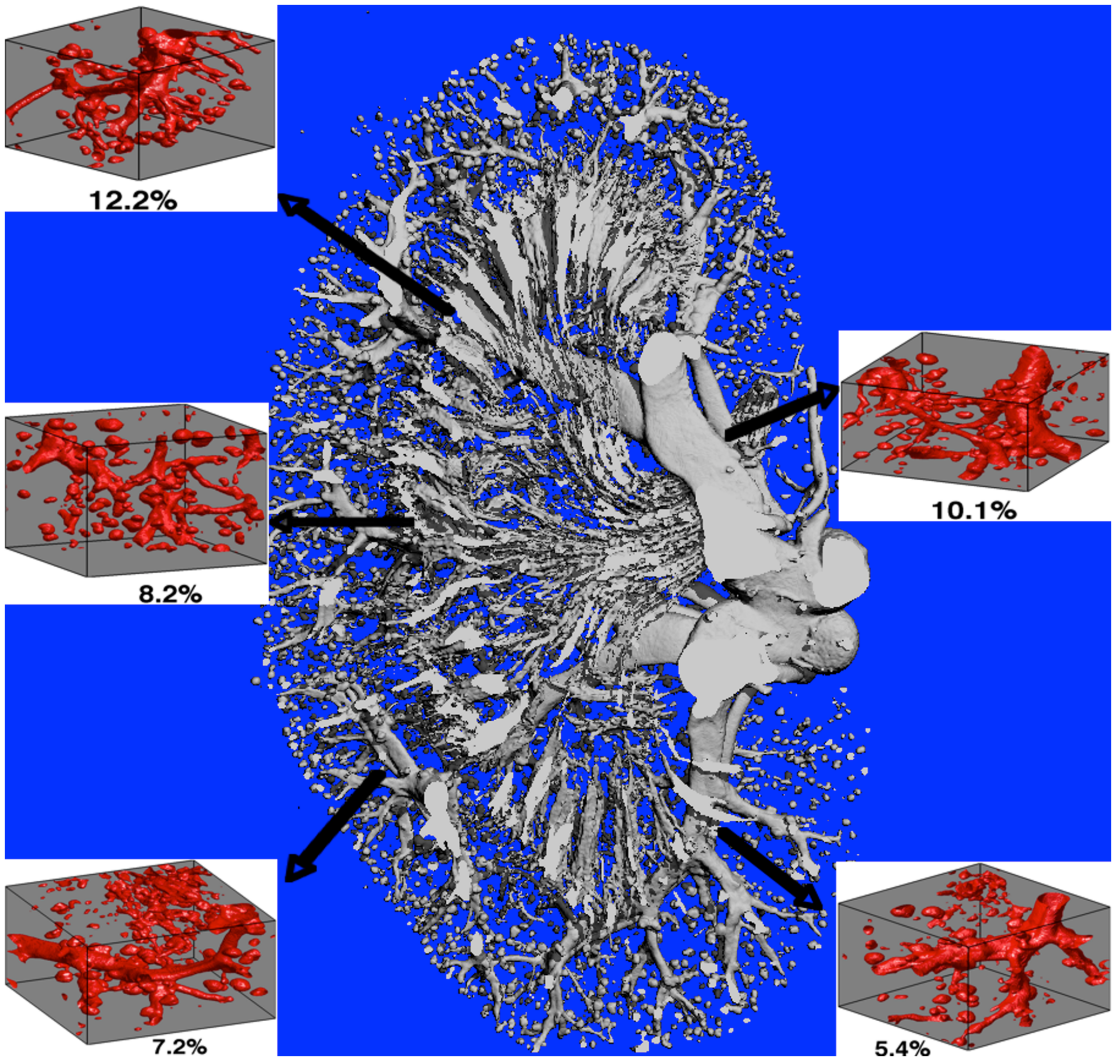
Kidney vascular structure extracted from micro-CT. Kidney vasculature extracted from micro-CT along with representative MR voxel-sized (1 mm^3^) microvascular models taken from different sections of the kidney vasculature with their respective vascular volume fractions. The existence of the bubble-like structures demonstrates the filling of glomeruli with Microfil but a higher resolution would be required to differentiate the individual capillaries.

**Figure 8:**
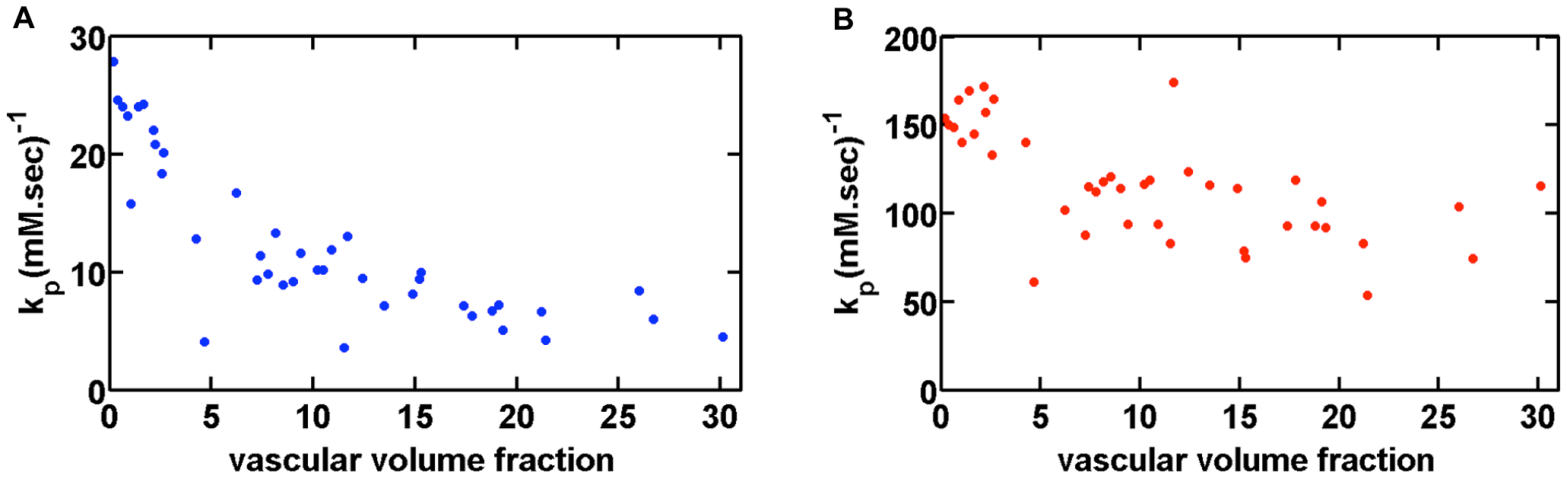
Computed k_p_ values for vascular structure extracted from micro-CT. (a) SE and (b) GE k_p_ values as a function of vascular volume fraction computed using the FPFDM for the kidney microvascular models (with vascular volume fractions >0.1%) shown in Fig. 7. SE k_p_ values ranged from 3.6–27.8 (mM–sec) ^−1^, and GE k_p_ values ranged from 53.8–174.3 (mM–sec) ^−1^. Above 5% volume fraction, the GE k_p_ values were relatively constant with a mean value of 103.3(mM–sec) ^−1^.

To further illustrate the versatility of the FPFDM, micro-CT images of perfused mouse kidney vasculature (1,242 slices with 1428×1012 matrix size and 5 µm isotropic voxels) were used as source data for multiple 1 mm^3^ vascular models with 200^3^ finite cubic perturbers, each 5 µm in size. Fig. 7 shows the entire extracted kidney vascular structure, with sample MR voxel-sized sub-structures and their respective vascular volume fractions. Fig. 8a and 8b shows the FPFDM derived SE and GE k_p_ values obtained from the slope of the ΔR_2_ and ΔR_2_^*^ dose response curves, respectively. These results are normalized to the vascular fractional volumes and were computed using B_0_ = 4.7T, D = 10^−5^ cm^2^/s, Δt = 0.2 ms, GE TE = 40 ms, SE TE = 80 ms, and a clinically relevant range of Δχ values ranging from 0 to 9.4×10^−8^, corresponding to a Gd-DTPA concentration ranging from 0 to 3.5 mM. In general, the SE and GE k_p_ values were highest for low vascular volume fractions and tended to decrease as the vascular volume fraction increased, with SE and GE k_p_ values ranging from 3.6–27.8 and 53.8–174.3 (mM–sec) ^−1^, respectively.

## Discussion

The FPFDM is a novel efficient computational tool combining features of the FP and FD techniques for calculating susceptibility-induced relaxivity changes for realistic simulated or imaging-based 3D vascular and cellular geometries that might be observed *in vivo*. The FPM can compute the induced magnetic fields around arbitrary microvasculature structures without necessitating any assumptions about the underlying vessel geometry [33]. Although the Fast Fourier transform (FFT) improves the computational efficiency of the FPM for computing magnetic field perturbations, the application of MC techniques for tracking proton diffusion through the tissue in order to derive the resulting relaxivity change reduces its computational efficiency. The replacement of the MC component of FPM with matrix-based FDM can increase the computational efficiency by computing the evolution of the discretized magnetization simultaneously [36]. The transition matrix *A* is either invariant or requires only partial updating for most tissues under consideration, further increasing the computing efficiency of matrix-based FDM that also benefit from optimized MATLAB packages for computations involving large matrices [36].

A Gaussian diffusion kernel convolution can also be used to model CA and water diffusion [39], [40], [48]. This approach is computationally more efficient than MC approaches, but limited to unrestricted water diffusion. Although non-Gaussian diffusion, a consequence of tissue structure that creates diffusion barriers and compartments, could be modeled by adding a kurtosis term to the kernel, it is not clear how this will affect the slower diffusion process observed in the restricted CA diffusion model [40]. Modeling restricted water diffusion using the MC method [33] or the Gaussian diffusion kernel approach [39], [40], [48] requires either incorporation of elastic collisions at membrane boundaries or neglecting proton diffusion steps that involve membrane crossing. Unlike the case of unrestricted water diffusion, using these later methods to model restricted water diffusion and/or water diffusion in complex tissue with different compartmental diffusion coefficients will require additional computations, thereby increasing the overall processing time. Given compartmental diffusion coefficients and membrane permeability values, the FPFDM can be used to model restricted water and CA diffusion and water exchange across compartments. For the FPFDM, including these additional structural features requires the computation of multiple versions of the diffusion transition matrix, *A*. Since *A* can be determined at the start of the simulation, a library of diffusion transition matrices, for a range of tissue structures, can be established to increase the computing efficiency. For example, computing a dynamic signal for the same structure only requires loading the transition matrix corresponding to the structure once from the library of diffusion transition matrices.

We validated the FPFDM in two ways. First, we replicated the vessel size dependence of ΔR_2_^*^ and ΔR_2_ (Fig. 2a) using identical simulation parameters to previously described MC and FP techniques [27], [33]. Next, we found excellent agreement for relaxivity from packed spheres across a range of packing densities and packing strategies using traditional MC technique versus FPFDM (Fig. 2b). The agreement between MC and FPFDM converges as the number of structures included in the average for the FPFDM increases (Fig. 2c). Unlike MC simulation, which tracks a large number of particles in the simulation or, equivalently, runs the same simulation many times to obtain an accurate average result, the FPFDM converges to the average result with only a few simulation runs. This behavior can be explained in the following way. In the MC simulation, a population of particles distributes in the whole system and the particles that encounter membranes within the complex tissue are only a small portion of all the particles such that the echo signal does not contain sufficient enough information about the tissue features that restrict diffusion. Hence, to solve this problem, more particles are considered in the simulation or, equivalently, the same simulation is run many times to obtain an accurate average result. In contrast, the FDFDM determines the diffusion transition matrix at the start of the simulation, which already contains the tissue structural information and results in a faster convergence of the average signal.

For a simplistic structure containing randomly oriented cylinders with a total of 18 different radii, the FPFDM, as compared to MC, reduced the computation time to calculate ΔR_2_^*^ values from 220 s to 140 s. For complex tissue structures, and under conditions of restricted water diffusion, the increase in computational efficiency afforded by the FPFDM will improve even further. In such cases, the MC method requires a larger number of spins and additional computation steps in order to converge and capture sufficient information about the tissue structure [49]. In contrast, for these more complex structures, the FPFDM does not require additional computing time and is not limited by restricted water diffusion [36].

The FPFDM has the potential for modeling nonstandard geometries that may better simulate cells and microvasculature *in vivo*. We computed relaxivities for simulated 3D cellular models consisting of packed spheres and ellipsoids (Fig. 3), and found greater relaxivity for packed ellipsoids over all volume fractions compared to the sphere packing. This suggests that the additional degree of freedom in spatial orientation for ellipsoids increases field perturbation heterogeneity. The greater orientational heterogeneity and packing density afforded by ellipsoids compared to spheres would seem to make ellipsoids better suited for simulating susceptibility-based contrast mechanisms of cellular structures.

Although simulations in this study are based on a simple 2-compartment model, at the expense of computational time, the same approach to model water diffusion and exchange can be used to model CA diffusion and transport across compartments, yielding a more realistic heterogeneous CA distribution within a voxel. This can be achieved by updating CA concentration for each voxel at each simulation time step using a CA diffusion transition matrix (*A_CA_*) calculated using appropriate CA diffusion coefficients and permeability across membranes from literature [50], [51]. For the purposes of this study, we assumed that the contrast agent equilibrates within each compartment over the time it takes to acquire each DSC-MRI image (1–2 seconds). Such an assumption is traditionally employed and consistent with current DSC-MRI analysis techniques.

We also computed the contrast agent concentration dependence of transverse relaxation rates for vascular trees. Traditionally, randomly oriented cylinders were used to investigate the influence of vascular properties (e.g. vessel size, vessel volume fraction) on relaxation rates. Fractal-based vascular trees better approximate the microvascular network in vivo, but this complex geometry with variable vessel rotation, size distribution, branching angles, and diameters of daughter vessels is very difficult to model and require high resolution to achieve structural details. Our results demonstrate the feasibility of using FPFDM for complex geometries, and suggest that although the generally accepted linear relationship between relaxation rate and CA concentration is reasonable, the proportionality constant *k_p_* depends upon the microvascular geometry, a finding that is consistent with previous studies [27], [33], [52]. The higher relaxation rate for vascular structures with greater range of branching angles is most likely due to the greater heterogeneity of vessel branch orientation with respect to B_0_ and their larger space occupancy which impacts the frequency offset of a larger volume compared to narrow branching angles that pack vessels in a small region. These preliminary computational results show marked *k_p_* heterogeneity across vascular networks, suggesting that further work is needed to better characterize the influence of vascular heterogeneity on DSC-MRI derived perfusion parameters in brain tumors. More systematic studies are underway for wide range of morphological, physiological and pulse sequence parameters to investigate these findings at the higher vascular volume fractions more typically encountered in DSC-MRI experiments, such as in grey matter or brain tumors.

The systematic evaluation of fractal-based vascular trees using the FPFDM could shed new insights into the relationship between DSC-MRI relaxation rate and vascular geometry. Furthermore, the use of fractal trees enables the application of well-established flow models [53]–[55] such that contrast agent kinetics and the associated DSC-MRI time series can be considered for each vascular structure. This would enable a more rigorous investigation of DSC-MRI-based voxel-wise measures of vessel size, transit time and flow distributions and oxygen extraction. Realistic 3D vascular and flow models could then be expanded to incorporate the extravasation of contrast agent and its subsequent diffusion around cells in the extravascular space. Such expansions would create a powerful framework with which to investigate DSC-MRI and susceptibility-based imaging methodologies in brain, tumors and other organs of the body.

The FPFDM also provides a computationally reasonable approach for simulating DSC-MRI derived transverse relaxation rates both in the presence and absence of CA extravasation, and restricted water diffusion induced by membrane permeability (Fig. 5 and Fig. 6). The results shown in Fig. 6 demonstrate that contrast agent leakage can lead competing T_1_ and T_2_^*^ effects as the CA traverses the extravascular extracellular space. For a given T_10_, the structure with smaller sized cells exhibited higher signal intensity recoveries as compared to that with larger sized cells. The compartmentalization of CA around the larger cells creates stronger magnetic field perturbations and greater relaxation rate changes (T_2_^*^ effects). In general, as T_10_ increases, T_1_ leakage effects will be more pronounced and may dominate any T_2_^*^ leakage effects, as is the case for the smaller-sized cells. In such cases, the characteristic signal overshoot may be observed (Fig. 6c). For the tissue structure with larger perturber sizes, the signal intensity exhibits less recovery due to the presence of substantial T_2_^*^ leakage effects (Fig. 6d–6f). As shown in Fig. 3b, cell density may also influence the shape of DSC-MRI signals, with the magnitude of T_2_^*^ leakage effects decreasing (and T_1_ leakage effects increasing) as the cell density increases. Consequently, DSC-MRI data from tumors with tightly packed, smaller-sized cells would likely present with pronounced T_1_ leakage effects (e.g. signal overshoot). Given the clinical importance of DSC-MRI signal recovery characteristics to help differentiate among tumor types [37], [56], a systematic *in silico* study of DSC-MRI signal recovery and its dependence on physiological, pulse sequence and physical parameters is currently under investigation.

Prior studies have shown the potential and value of incorporating image-based vascular structures into susceptibility simulations [39]. Similar to these previous studies we sought to demonstrate the versatility of the FPFDM by determining the dose-response of relaxation rates for vascular structures derived from *ex vivo* micro-CT scans of perfused kidney vasculature. The dose-response curves from MRI voxel-sized regions of the kidney vasculature were used to determine the distribution of vascular susceptibility calibration factors, k_p_, within the kidney. For vascular volume fractions up to 30%, k_p_ values were very heterogeneous (Fig. 8), with decreased heterogeneity for vascular volume fractions greater than 5%. The k_p_ decreased over vascular volume fractions between 0 and 5% with a slower decrease above 5%, consistent with a previous study in rodent brain that found grey matter k_p_ to be nearly twice that of tumor [34]. It should be noted that the kidney microvascular structure presented in this study is limited by the spatial resolution of the micro-CT data. With a 5 mm, resolution individual capillaries could not be resolved and capillary dense regions, such as in the glomeruli, present as a single large perturber. The differentiation and inclusion of these capillaries will likely influence the overall k_p_ heterogeneity across voxels for both SE and GE computations. For the purposes of this study, this example illustrates the ability of the FPFDM to explore susceptibility contrast in tissue structures acquired using *ex vivo* imaging modalities. As the FPFDM only requires that structures consist of a digital format it could accept structural input from any imaging modality (e.g. optical, CT, electron microscopy, MRI).

One of the limitations of the FPM is the use of FFT to calculate the spatial convolution of the vascular structure with the finite perturber magnetic field perturbation. As demonstrated in [33] the resulting field perturbation is equivalent to the field perturbation from a periodic array of the tissue structure under consideration. Although realistic tissue structures extend beyond the boundary of the simulation space, which introduces a “boundary problem”, we used zero-padding of the tissue structure to avoid additional field perturbation at the boundaries from the neighboring array. The padding size to eliminate boundary field effects depends on the perturber size and the tissue structure. Here we used a zero-pad size of one-tenth of the simulation box, since the field perturbation changes we observed by using higher zero-pad sizes were negligible.

The FPM was designed to compute the magnetic field changes from a single finite perturber convolved with a digitized tissue structure array, and hence this approach cannot be used for arbitrary magnetic susceptibility distributions. While methods capable of computing arbitrary susceptibility distributions are more comprehensive and should be explored [57], [58], it is typically assumed that contrast agent instantaneously distributes within each tissue compartment (e.g. intravascular and extravascular extracellular space) at each imaging time point. Accordingly, the FPFDM is a practical approach to compute field perturbations arising from tissue structure with only a few susceptibility compartments, such as the intravascular, intracellular, and extravascular extracellular spaces.

The sampling of tissue structures at higher resolution increases the computational accuracy of the FPM but it comes at the expense of computational time. Such increases in resolution would also add to computational time needed to compute the MR signal using the FDM. This is particularly true if a need arises to reduce the simulation time step (Δ*t*) due to increased resolution or decreased perturber size (Δ*x*), in order to satisfy the constraint that the jump probability (see Eq. 6) should be ≤1/6. This is because when the number of spins leaving a given node exceeds the number that was present, the FDM becomes unstable [35]. With the parallel high-performance computing techniques we previously developed [36], we are exploring ways to increase the computational efficiency of the FPFDM at higher resolutions so that we can more accurately characterize fine tissue microstructure across a broader range of structural dimensions (e.g. a few microns up to a hundreds of microns).

## Conclusion

The FPFDM is an alternative computational tool for efficiently modeling susceptibility induced MR signal relaxation from complex perturber geometries. In general, the proposed FPFDM could be used to investigate the influence of realistic tissue microstructure on any susceptibility based contrast mechanism such as vessel size imaging, BOLD contrast, single cell imaging, and quantitative susceptibility mapping. Currently, the proposed method is being utilized to assess the influence of geometrical, morphological and physiological parameters of microvessels and cells on susceptibility induced MR relaxation rate changes. Such studies should shed new insights into DSC-MRI contrast mechanisms and enable the systematic evaluation of how acquisition and analysis methods influence the measurement of reliable perfusion parameters in brain and tumor tissue.
